# Further Meditations on Gout

**Published:** 1919-08-23

**Authors:** Chas. Mercier


### THE EDITOR'S LETTER-BOX.

**[Correspondence on all subjects is invited, but we cannot in any way be responsible for the opinions expressed by our correspondents, who must give their name and address as a guarantee of good faith, but not necessarily for publication. Correspondents are reminded that brevity of style and conciseness of statement greatly facilitate early insertion.]**

### FURTHER MEDITATIONS ON GOUT.

*To the Editor of* The Hospital.

Sir,—I have on previous occasions sent you some meditations written during an attack of this disease, and since that occasion I have had no opportunity till now of meditating on it in similarly favourable circumstances.

During the hot weather that has prevailed here for the last ten days, my usual habit of abstemiousness in food and drink has been pushed almost to the point of abstinence, and in spite of this, or perhaps in consequence of this, I have once more received a visit from my hereditary enemy.

The visitation is the more remarkable since I have given him twice recently invitations of which he has not cared to avail himself. Circumstances prevented me, much to my sorrow, from witnessing in person the presentation of a memorial to my excellent friend Professor Osler; but as I could not be present except in spirit, I did what I could to honour the revered Regius Professor by drinking his and Lady Osler's healths in as much port as seemed appropriate for such an important occasion.

On Peace night I issued a still more defiant challenge. After washing down an excellent dinner with a sufficiency of champagne, I drank in port to the separate and individual healths of Marshal Foch, the Sovereigns and Presidents of the Allied and Associated Powers, their several Prime Ministers, and the Commanders of the victorious forces by sea and by land. I terminated the evening by drinking damnation to the ex-Kaiser and all his ex-satellites, and finally drained a bumper to the deeper damnation of the Pope for looking on with bland unconcern while his priests were being murdered and his nuns were being violated by the Germans. Shades of Gregory the Great and Innocent III.!

The port, Sir, was excellent. Not, of course, as good as College port or City Company port, but it was good. None of your decolorised and attenuated tawny fluid, which has yielded up all its strength and virtue to the insensible cask in which it dissipated its colour, but good rich fruity port, with as much and as noble a body in it as the ground under Westminster Abbey, and with the rich colour and the inward fiery glow of the carbuncle. Talk not to me of the washy insipid diamond, or the pigeon-blooded ruby! Like champagne and claret, they are very suitable for women, but for men your only wear is the glowing carbuncle—pity that he is always cut *en cabochon*, and never has a chance to display his full magnificence and splendour! The port, if not all that port can be, was all that can reasonably be expected outside of a City Company's Hall or the Common Room of a College, and yet, or perhaps consequently, I woke next morning like the Assyrian, my hallux unswollen, my slumbers unspoilt, ready to forgive even teetotallers, since by their abstinence they do at least refrain from forcing still higher the present unreasonable price of port. So we see that there is some sort of goodness in things evil if men would diligently search it out; and even a teetotaller has a certain negative merit. The eye of Goodness, shrewdly observes Mr. Sterne, discerneth all things.

Your more robust readers may perhaps consider that in bestowing even this meed of praise upon the teetotaller, I am verging upon the maudlin. It may be so; but it is at least a refutation of the foul libel that attributes irritability and ill-temper to the sufferer from gout. A man who can see even negative merit in a teetotaller may be maudlin, but so far from being irritable or ill-tempered, he must have a disposition little short of angelic. Perhaps I am not the person who ought to say it, but if I don't, who will?

One of Scott's characters declared that she was fasting from everything but sin and Bohea. I may fairly claim to be still more abstemious, for I abjure even one—the first—of these indulgences. "Tears are my daily food," said the stricken patriarch. Except on the two occasions mentioned, weak tea has been my only beverage for the last few months, and the consequence is that my foot is like that of the Emperor Severus in Leich's classical picture. It is time to try what port will do to drive the gout away. I do not know what the recent suppositions are as to the origin of gout, but whatever they are they must take into account all occurrences like this.—I am, Sir, your obedient servant,

Chas. Mercier.

